# A Genetic Incompatibility Accelerates Adaptation in Yeast

**DOI:** 10.1371/journal.pgen.1005407

**Published:** 2015-07-31

**Authors:** Duyen T. Bui, Elliot Dine, James B. Anderson, Charles F. Aquadro, Eric E. Alani

**Affiliations:** 1 Department of Molecular Biology and Genetics, Cornell University, Ithaca, New York, United States of America; 2 Department of Biology, University of Toronto, Mississauga, Ontario, Canada; Washington University School of Medicine, UNITED STATES

## Abstract

During mismatch repair (MMR) MSH proteins bind to mismatches that form as the result of DNA replication errors and recruit MLH factors such as Mlh1-Pms1 to initiate excision and repair steps. Previously, we identified a negative epistatic interaction involving naturally occurring polymorphisms in the *MLH1* and *PMS1* genes of baker’s yeast. Here we hypothesize that a mutagenic state resulting from this negative epistatic interaction increases the likelihood of obtaining beneficial mutations that can promote adaptation to stress conditions. We tested this by stressing yeast strains bearing mutagenic (incompatible) and non-mutagenic (compatible) mismatch repair genotypes. Our data show that incompatible populations adapted more rapidly and without an apparent fitness cost to high salt stress. The fitness advantage of incompatible populations was rapid but disappeared over time. The fitness gains in both compatible and incompatible strains were due primarily to mutations in *PMR1* that appeared earlier in incompatible evolving populations. These data demonstrate a rapid and reversible role (by mating) for genetic incompatibilities in accelerating adaptation in eukaryotes. They also provide an approach to link experimental studies to observational population genomics.

## Author Summary

In nature, bacterial populations with high mutation rates can adapt faster to new environments by acquiring beneficial mutations. However, such populations also accumulate harmful mutations that reduce their fitness. We show that the model eukaryote baker’s yeast can use a similar mutator strategy to adapt to new environments. The mutator state that we observed resulted from an incompatibility involving two genes, *MLH1* and *PMS1*, that work together to remove DNA replication errors through a spellchecking mismatch repair mechanism. This incompatibility can occur through mating between baker’s yeast from different genetic backgrounds, yielding mutator offspring containing an *MLH1-PMS1* combination not present in either parent. Interestingly, these offspring adapted more rapidly to stress, compared to the parental strains, and did so without an overall loss in fitness. DNA sequencing analyses of baker’s yeast strains from across the globe support the presence of incompatible hybrid yeast strains in nature. These observations provide a powerful model to understand how the segregation of defects in DNA mismatch repair can serve as an effective strategy to enable eukaryotes to adapt to changing environments.

## Introduction

DNA mismatch repair (MMR) acts primarily during DNA replication in prokaryotes and eukaryotes to correct DNA polymerase misincorporation errors that include base substitutions, frameshift mutations, and insertions/deletions [1–3]. In *S*. *cerevisiae* MutS homolog (MSH) heterodimers can track with the replication fork to recognize and bind to DNA mismatches [4,5]. MSH-marked repair sites are recognized primarily by the MutL homolog (MLH) heterodimer Mlh1-Pms1. The resulting ternary complex interacts with downstream excision factors such as Exo1 to remove the newly replicated DNA strand where the misincorporation event had occurred.

Defects in MMR result in the accumulation of deleterious mutations and an overall loss in fitness (e.g. [6]). Interestingly, studies in microbes have shown that the mutation rate per base pair is inversely proportional to genome size, and that changes from the wild-type rate are selected against [7–10]. However, approximately 10% of natural *E*. *coli* isolates display a mutator phenotype with 1–3% displaying defects in the MMR pathway [11–12]. The finding that a high mutation rate is typically selected against, but that some bacterial isolates can be observed in populations that are mutators, suggests that mutators may play an important role in adaptive evolution [11, 13–20]. One explanation for this observation is that mutators have an increased likelihood of acquiring the first adaptive mutations within a population. However, such a strategy is not sustainable due to the accumulation of deleterious mutations that ultimately outweigh beneficial mutations. Bacteria appear to solve this problem through horizontal transfer; mismatch repair genes are exchanged between genomes at higher than average rates, which is likely due to the hyper-recombination phenotypes exhibited by MMR-deficient strains [14].

Do eukaryotes also regulate MMR functions to adapt to new selective pressures? Previously Thompson et al. [21] showed that diploid baker’s yeast lacking the *MSH2* MMR gene display an adaptive advantage when competed against diploid non-mutators. However, this advantage was not seen in haploids. Previously we hypothesized that MMR function could be modulated in eukaryotes through negative epistatic interactions [22]. This hypothesis was based on experiments in which we mated two *S*. *cerevisiae* strains, S288C and SK1, which show 0.7% sequence divergence, and identified one *MLH* genotype, S288c *MLH1*-SK1 *PMS1*, that conferred mutation rates 100-fold higher than wild type in an assay in which *mlh1* and *pms1* null strains display a 10,000-fold higher rate [22]. The S288c *MLH1*-SK1 *PMS1* combination was defined as ‘incompatible’, while the other three combinations, which did not display a mutator phenotype, were labeled ‘compatible’. A single nucleotide polymorphism (SNP) in *PMS1* combined with a single SNP in *MLH1* were primarily responsible for the incompatibility [22].

**Fig 1:**
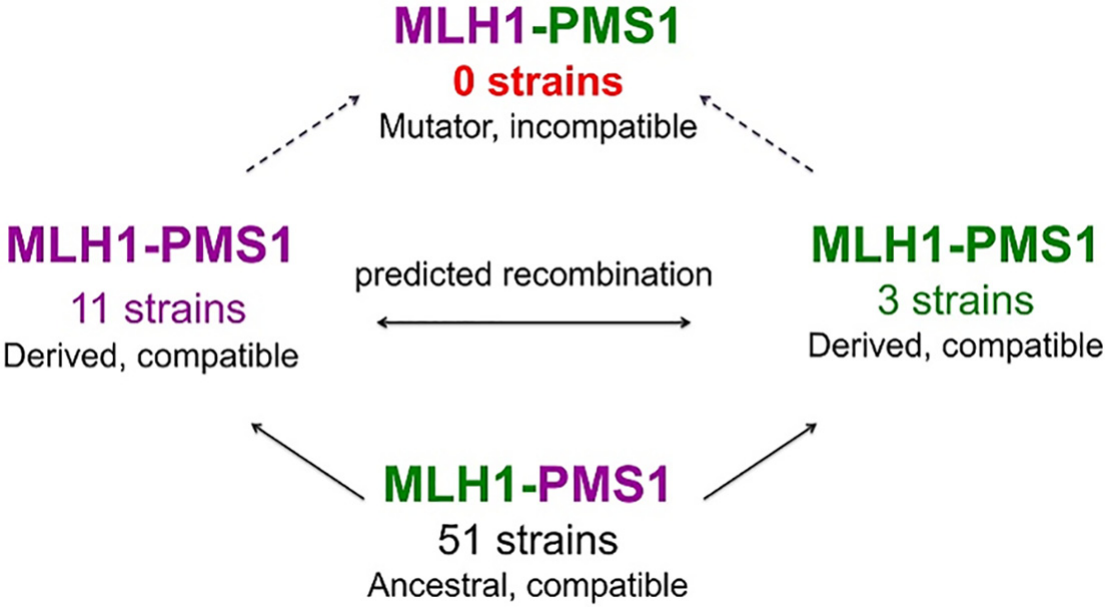
A model for how MMR incompatible populations arise in nature [22,27]. In this cartoon, a common ancestor bearing the Mlh1 Gly 761 and Pms1 Arg 818/822 alleles can sustain mutations, neutral or beneficial, that give rise to the derived S288c (purple, Asp 761, Arg 818/822) and SK1 (green, Gly 761, Lys 818/822) group strains. Mating between the derived strains can yield an allele combination (Mlh1 Asp 761, Pms1 Lys 818/822) that had not been selected for function, leading to a negative epistatic interaction and a mutator phenotype. Sequencing analysis of a 32-kb region in the derived groups provided evidence for recombination between the two, supporting the idea that these two groups can meet in nature, exchange genetic information, and form a hybrid mutator [22].

Dobzhansky and Muller proposed a model to explain how hybrid incompatibilities can arise without causing defects within parental strains or species [23–26]. As described previously [22,27], the evolution of the S288c *MLH1*-SK1 *PMS1* MMR incompatibility (Fig 1) fits this model. Mating of S288C and SK1, followed by sporulation and segregation of gene variants within progeny, creates an S288c *MLH1*-SK1 *PMS1* genotype that shows negative epistasis (Fig 1; [27]). Such negative epistasis is similar to the interactions thought to underlie hybrid incompatibility between established [28–32] or incipient species [33].

DNA sequence analyses of natural and laboratory yeast strains indicated that S288c and SK1 strains have mated naturally [22]. This finding suggests that incompatible combinations were likely to have been created in nature but were not maintained due to losses in fitness associated with defects in MMR (e.g. [3,22,27]). We hypothesize that negative epistasis involving MMR gene variants provides a transient advantage critical for adaptive evolution. To test this idea we constructed isogenic compatible and incompatible *MLH1-PMS1* strains and subjected them to adaptive evolution in high salt. We found that incompatible populations adapted more rapidly to high salt than compatible strains without displaying an apparent fitness cost. Furthermore, we show that mutations in *PMR1* were causative for high salt resistance in incompatible populations. Interestingly, mutations in this same gene, *PMR1*, subsequently arose in compatible populations though at a slower rate. Together these observations demonstrate an experimentally validated role for genetic incompatibilities in accelerating adaptation to environmental challenges in eukaryotes.

## Results

### *MLH1-PMS1* incompatible strains display a fitness advantage when evolved in high salt

We tested if the negative epistasis phenotype seen in yeast bearing the S288c *MLH1*-SK1 *PMS1* genotype confers an adaptive advantage during stress. This study was initiated by constructing isogenic compatible and incompatible *MLH1-PMS1* strains that displayed, prior to adaptation, similar fitness levels in YPD and YPD + 1.2 M NaCl media as measured in growth and competition assays (Materials and Methods; S1 Table and S1 Fig). We assessed the mutator phenotype of compatible and incompatible strains using the *lys2-A*_*14*_ reversion assay. Compared to SK1 *MLH1*-SK1 *PMS1* compatible strains, S288c *MLH1-*SK1 *PMS1* incompatible strains showed increased reversion rates similar to that seen in previously constructed incompatible strains (100 to 120-fold higher than S288c *MLH1*-S288c *PMS1*; S2 Table; [22]).

Compatible and incompatible lines were analyzed for adaptation to high salt conditions by growing them in YPD media containing 1.2 M NaCl as described in the Materials and Methods. In YPD media both compatible and incompatible lines completed 8–9 generations per transfer. In YPD + 1.2 M NaCl media both compatible and incompatible lines completed ~5.5 generations after the first transfer. A steady rise in the number of generations completed, from ~6.0 to ~6.8, was seen after Transfers 2 to 20. Cultures obtained after 7 (~50 generations), 10 (~70 generations), and 16 (~120 generations) transfers showed the maximal fitness advantage gained by incompatible lines. Growth rate was determined by measuring the OD_600_ of cultures every two hours following dilution into YPD + 1.2 M NaCl. Pair-wise competition experiments were performed by mixing equal amounts of cells obtained from randomly chosen incompatible and compatible cultures. The proportion of cells in each culture was determined at T = 0 and T = 24 hrs following mixing (Materials and Methods).

**Fig 2:**
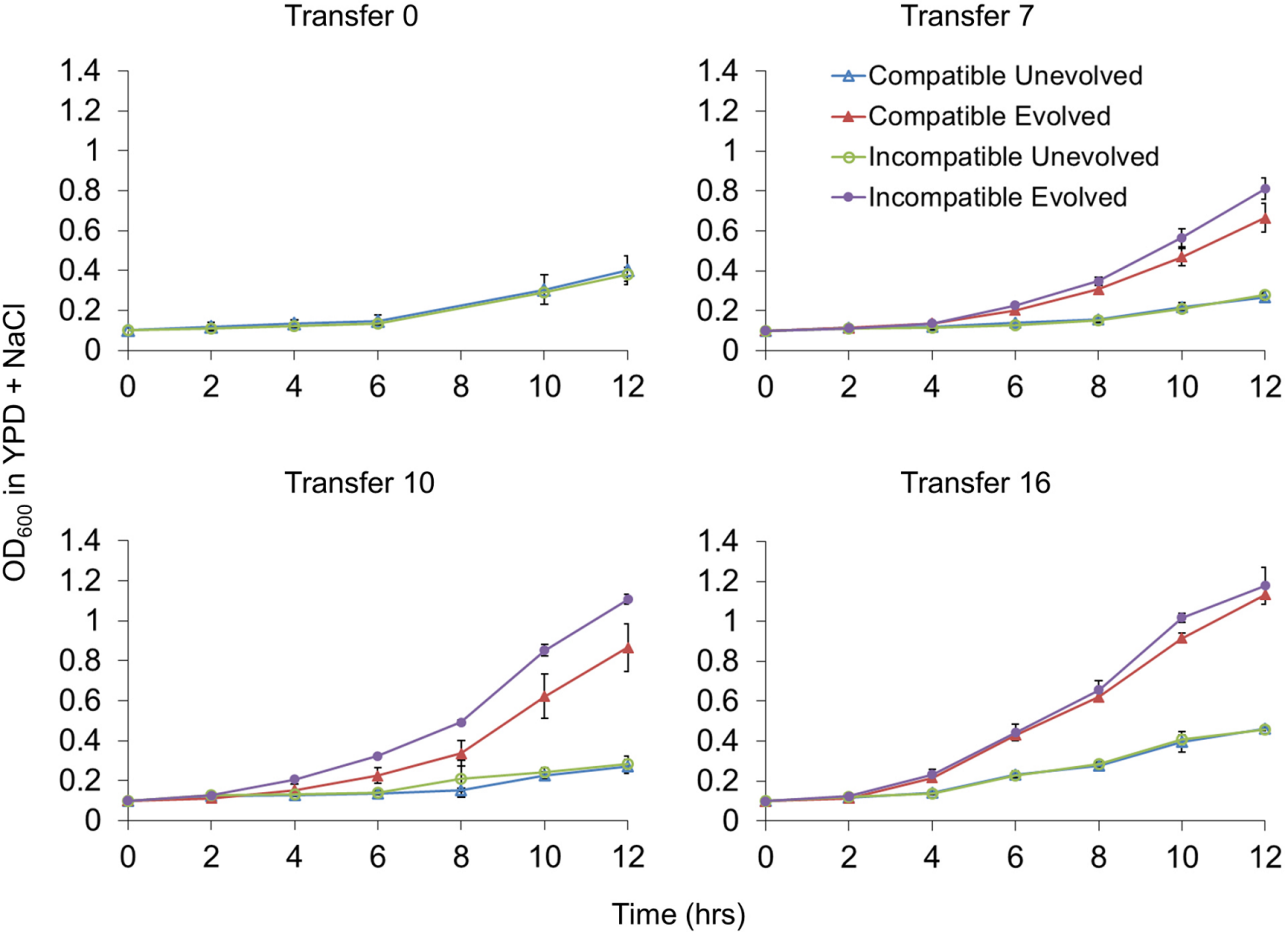
Incompatible strains display a fitness advantage in high salt media. Independent cultures of compatible (*kMLH1-kPMS1*, EAY3242) and incompatible (*cMLH1-kPMS1*, EAY3236) strains were grown for up to Transfer 16 (~ 2 x 10^7^ cells per transfer) in YPD (unevolved) or YPD + 1.2 M NaCl (evolved). Cultures at the indicated transfers were diluted to an OD_600_ of 0.1 (~ 2 x 10^7^ cells per transfer) in YPD + 1.2 M NaCl and monitored for growth for 12 hrs. A representative experiment involving three replicates for each genotype is shown. Mean OD_600_, +/- standard deviation, is presented for each time point. See Materials and Methods for details.

We assessed the growth of isogenic compatible and incompatible lines in YPD and YPD + 1.2 M NaCl. These lines could be distinguished from each other in experimental cultures because they contained different antibiotic resistance markers (*KANMX*, resistance to G418, and *NATMX*, resistance to nourseothricin) linked to the *MLH1* locus (S1 Table). The markers could be switched between compatible and incompatible strains without an effect on fitness in growth and competition assays, indicating that the antibiotic markers did not confer a selective advantage. As shown in Fig 2, incompatible lines displayed a faster growth rate in YPD + 1.2 M NaCl after Transfer 7 (~50 generations). This advantage was more apparent after Transfer 10 (~70 generations; p < 0.0001, n = 25), but was not seen after Transfer 16 (~120 generations; p >0.05; n = 8).

In addition to direct growth measurements, we measured fitness in competition assays in which cells from randomly chosen compatible and incompatible evolved lines were mixed together at an approximately 1:1 ratio. These competitions involved cells adapted in YPD + 1.2 M NaCl that had undergone the same number of transfers. After mixing, the lines were grown in YPD or YPD + 1.2 M NaCl for 24 hrs (seven generations) and the proportion of each type was determined (Materials and Methods). As shown in Table 1 and S2 Fig, neither compatible nor incompatible lines showed a competitive advantage in YPD media. In YPD + 1.2 M NaCl media, neither of the two lines showed a competitive advantage after Transfer 7. However, incompatible lines displayed a competitive advantage in this media after Transfer 10 (p = 0.0031), with an average fitness advantage of 16% over compatible lines (Table 1). This advantage was lost after Transfer 16 (p = 0.39). Together, these data indicate that a MMR incompatibility generated by recombination involving naturally occurring variants in *PMS1* and *MLH1* can lead to an elevated rate of occurrence of adaptive mutations, and thus accelerate adaptation in a eukaryote.

**Table 1:** Fitness of incompatible relative to compatible strains following competition.

Fitness, *w* +/-SEM, (n)			ANOVA
Transfer	YPD	YPD+NaCl	(p-value)
0	0.99 ± 0.02 (10)	0.95 ± 0.01 (10)	0.1417
7	0.98 ± 0.02 (12)	1.01 ± 0.02 (12)	0.3806
10	0.99 ± 0.01 (16)	1.16 ± 0.04 (16)	0.0031 *
16	0.96 ± 0.03 (6)	0.90 ± 0.06 (6)	0.3905

Independent cultures of compatible (*kMLH1-kPMS1*, EAY3242) and incompatible (*cMLH1-kPMS1*, EAY3236) strains were grown for the indicated number of transfers in YPD + 1.2 M NaCl. Fitness values were separately determined after competition experiments in which evolved cultures were randomly mixed at a 1:1 ratio and grown for an additional 24 hours in YPD or YPD + 1.2 M NaCl (see examples of the raw data in S2 Fig). Fitness (*w*) [34,35] was calculated as *w* = ((p_t_/q_t_)/(p_o_/q_o_))^1/t^, where t equals the number of generations after 24 hrs of competition (7 generations), p_o_ and q_o_ are the number of incompatible and compatible cells, respectively at 0 hrs, and p_t_ and q_t_ are the number of incompatible and compatible cells, respectively, at 24 hrs. n is the number of unique competitions performed for each data set. One-way ANOVA [36] was used to test whether mean fitness values are different in YPD + 1.2M NaCl vs. YPD.

### The fitness advantage seen in incompatible strains depends on mutation supply

How can we explain the temporal rise in fitness advantage seen in incompatible versus compatible lines? The most straightforward explanation is that the supply of mutations in the incompatible lines is higher than in the compatible lines, thus providing a greater likelihood for obtaining beneficial mutations that reach a high enough frequency to be selected and maintained in a population (e.g. [20,21,37]). The mutation supply available is a function of the mutation rate and the population size (N). To test this idea, we lowered the mutation supply by reducing the number of cells (and thus population size) per transfer in YPD-1.2 M NaCl by ten-fold to ~2 x 10^6^ cells per transfer. We then performed cell growth and competition assays. In this experiment we were unable to observe a statistically significant advantage (p> 0.05) in fitness for the incompatible strains even though the number of generations completed, 70 to 75 after Transfer 10, were similar. In this experiment *w* = 0.99 +/-0.04 (SEM, n = 4) in YPD, and *w* = 0.96 +/- 0.02 (SEM, n = 4) in YPD-1.2 M NaCl (see also S3 Fig). These observations thus support the premise that mutation supply is critical to achieve the fitness advantage seen in incompatible strains.

Why was a fitness advantage in high salt seen in incompatible strains at Transfer 10 but not at Transfer 16? One possibility is that compatible populations adapt more slowly due to a lower mutation supply, but eventually obtain beneficial mutations that are selected for and maintained in the population. Alternatively, and/or in conjunction, incompatible populations accumulate a greater number of deleterious mutations and lose fitness over time. As shown in Table 1 and S2 Fig, the fitness of compatible and incompatible cultures was similar in YPD media even after 16 transfers, when the fitness advantage for incompatible lines in YPD + 1.2 M NaCl media was no longer apparent. Together these observations and the mutation supply experiments presented above and S3 Fig indicate that the speed by which compatible and incompatible populations adapt is dependent on the mutation supply rate, which is higher in the incompatible strains.

### Mutations in *PMR1* were identified in both compatible and incompatible lines grown in YPD + 1.2 M NaCl

**Fig 3:**
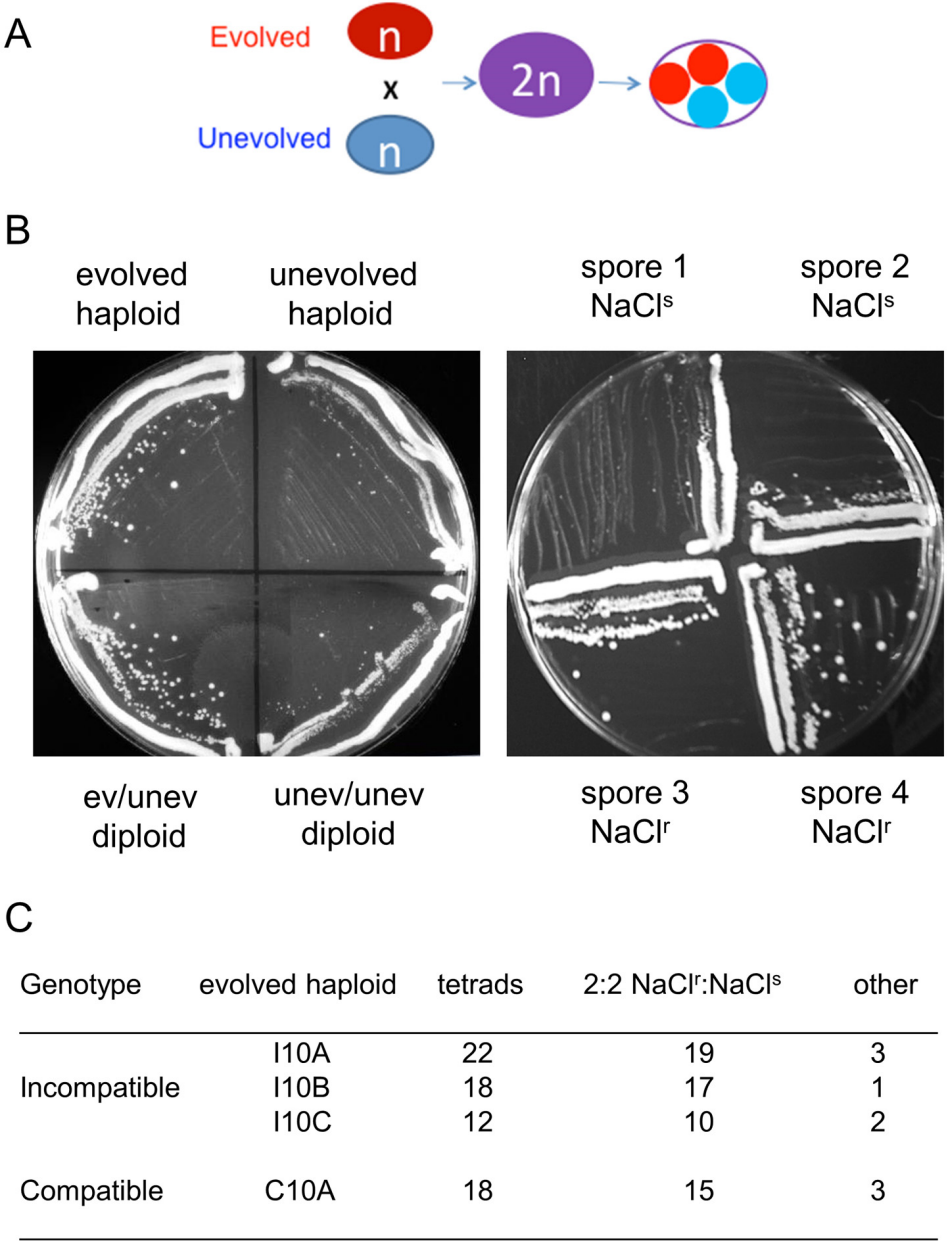
A single locus is likely responsible for high salt adaptation in compatible and incompatible strains. A, High salt resistant clones (red) isolated from evolved compatible and incompatible cultures were crossed to an unevolved strain (blue). Diploids were selected and streaked onto YPD + 1.2 M NaCl plates to determine if NaCl^r^ in the evolved strain was dominant or recessive. Subsequently, diploids were sporulated and tetrad dissected, and spore clones were analyzed for resistance to NaCl. B. Left panel. An evolved NaCl resistant clone showing a dominant phenotype. Growth of the indicated haploid and diploid strains on YPD + 1.2 M NaCl plates is shown. Right panel, example of 2:2 NaCl^r^:NaCl^s^segregation. Growth on YPD + 1.2 M NaCl plates is shown for the spore clones of a single tetrad obtained by mating an evolved NaCl^r^ clone to an unevolved strain. C. The indicated incompatible and compatible evolved NaCl^r^ clones were each mated to an unevolved haploid strain and analyzed for segregation of NaCl resistance in tetrad analysis. For each mating the vast majority displayed 2:2 segregation of resistance to sensitivity. In total nine tetrads deviated from this pattern (“other” category) with eight showing 3:1 or 1:3 segregation and one showing 4:0 segregation.

Are mutant alleles of the same genes responsible for salt resistance in incompatible and compatible populations? We answered this question by isolating salt-resistant clones (one per line) from independent incompatible and compatible lines. NaCl resistance in evolved strains can be easily phenotyped on YPD + NaCl plates because they grow to larger colony sizes relative to unevolved strains (Fig 3B, left panel). To determine the complexity of the NaCl resistant phenotype, we mated these clones (primarily from transfer 10) to unevolved strains of the opposite mating type (Fig 3) to form diploids. While most (five of eight tested) of the diploid strains were sensitive to NaCl, indicating recessive transmission, three of the eight displayed a semi-dominant phenotype (example in Fig 3B, left panel). Four diploids created by mating evolved and unevolved strains were then sporulated and phenotyped for salt resistance. Interestingly, all four strains displayed primarily a 2 NaCl^r^:2 NaCl^s^ segregation phenotype on YPD + NaCl plates, indicating that a single locus in the evolved strain was causative. At least 18 NaCl^r^ and 18 NaCl^s^ spore clones derived from each of the four matings were pooled separately and subsequently analyzed by whole-genome sequencing using a bulk segregation strategy (Materials and Methods). As shown in Table 2, only one to three mutations were identified in each of the four clones. Interestingly, in all four clones only one locus, *PMR1*, displayed strong linkage, as measured in sequence read counts, to the NaCl^r^ phenotype (p < 10^−5^ for all linkages to *PMR1*). As described in further detail below, *PMR1* encodes a membrane-bound P-type Ca^2+^ dependent ATPase involved in transporting Mn^2+^ and Ca^2+^ into the Golgi [38]. In subsequent paragraphs we describe a detailed analysis of *pmr1* mutants identified in evolved cultures, with the goal of explaining the genetic basis of adaptation to a defined stress, in this case, high salt.

**Table 2:** Whole genome sequencing indicates *PMR1* linkage to NaCl resistance.

				NaCl^r^ pool		NaCl^s^ pool		
Line	SGD locations	*pmr1* mutation	WT/SNP	WT	SNP	WT	SNP	linkage
I10A incompatible	chrVII:190010	T459A	A/T	1	58	54	1	Yes
	chrVIII:555937		A/G	29	13	29	35	No
I10B incompatible	chrII:48117		C/T	42	51	29	28	No
	chrVII:190057	T412C	A/G	0	91	50	0	Yes
I10C incompatible	chrIV:13937		G/A	50	40	55	46	No
	chrVII:190467	T2G	A/C	0	14	91	0	Yes
	chrXIII:908203		G/A	11	18	86	61	No
C10A incompatible	chrVII:188442	C2027T	G/A	0	70	104	6	Yes

The indicated evolved clones obtained from Transfer 10 were each mated to an unevolved compatible strain (EAY3241 for mating to incompatible evolved, EAY3191 for mating to compatible evolved). The resulting diploids were sporulated and tetrad dissected and germinated spore clones were analyzed for growth on YPD containing 1.2 M NaCl. For each mating at least 18 NaCl^r^ and 18 NaCl^s^ spore clones were separately pooled. The resistant and sensitive pools were then analyzed by whole genome sequencing (Materials and Methods). The sequence for the indicated position is shown for the unevolved reference (WT) and the evolved strains (SNP), followed by the number of WT and SNP reads detected in each pool. Using the Fisher Exact test, all SNPs we found that were defined as linkage differ from random segregation with a p value <0.0001.

**Fig 4:**
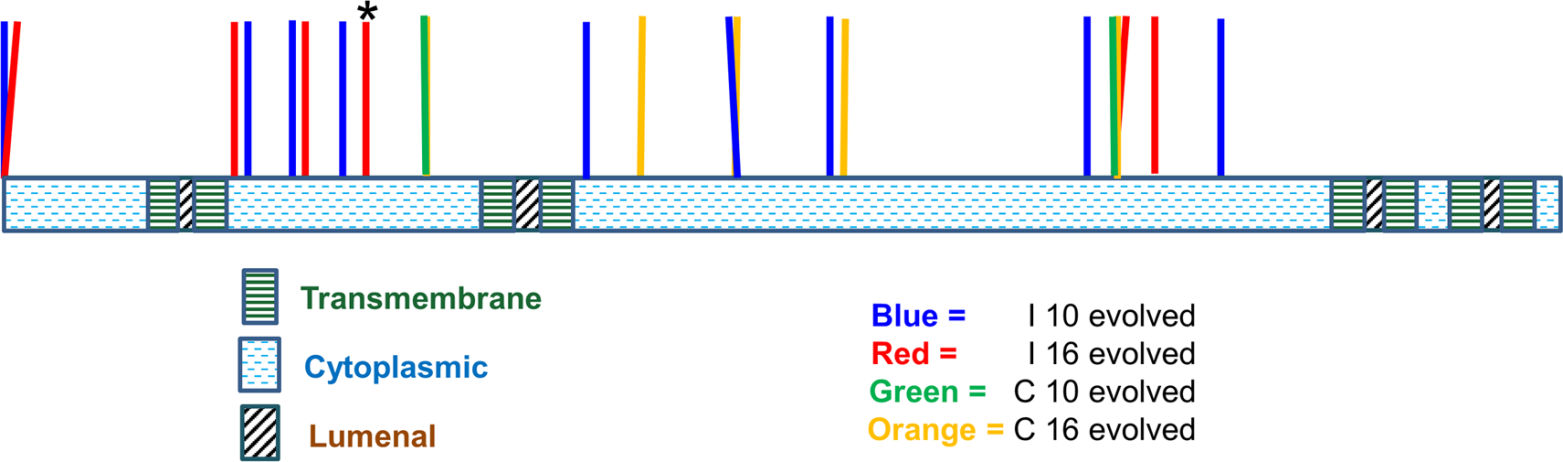
Location of *pmr1* mutation alleles found in evolved strains. 21 *pmr1* mutations (see Table 3 for the exact locations) identified in this study were mapped onto the Pmr1 structure predicted by Uniprot (Materials and Methods). * indicates the presence of a frameshift mutation.

We sequenced in total 37 clones obtained from independent compatible or incompatible lines grown in YPD +1.2 M NaCl. Twelve of these were subjected to whole genome sequencing. For 25 clones Sanger sequencing was performed on the PCR-amplified *PMR1* locus. As shown in Table 3 and Fig 4, 21 different mutations in *PMR1* were identified in the 37 clones that mapped to the predicted cytoplasmic domain of Pmr1. While most mutations resulted in amino-acid substitutions in the 950 amino acid Pmr1 protein sequence, two involved start codon disruptions, and one was a frameshift mutation predicted to disrupt the reading frame beginning at amino acid 220.

**Table 3:** *pmr1* mutations identified in this study.

Evolved clone		Sequencing method	*pmr1* mutation	Amino acid change
C10	A	WGS	C2027T	A676V
C10	B	WGS	None	n/a
C10	C	WGS	None	n/a
C10	D	Sanger, *PMR1* PCR	None	n/a
C10	E	Sanger, *PMR1* PCR	None	n/a
C10	F	Sanger, *PMR1* PCR	None	n/a
C10	K	Sanger, *PMR1* PCR	None	n/a
C10	L	Sanger, *PMR1* PCR	None	n/a
C10	M	Sanger, *PMR1* PCR	None	n/a
C10	N	Sanger, *PMR1* PCR	A778C	T260P
C16	A	WGS	C2027T	A676V
C16	G	WGS	T-220A*	
C16	H	Sanger, *PMR1* PCR	G1348T	D450Y
C16	I	Sanger, *PMR1* PCR	G1121T	G374V
C16	J	Sanger, *PMR1* PCR	C1532T	S511F
C16	N	Sanger, PMR1 PCR	A778C	T260P
I10	A	WGS	T459A	C153→stop codon
I10	B	WGS	T412C	S138P
I10	C	WGS	T2G	start codon disruption
I10	D	WGS	T2213C	F738S
I10	E	WGS	A1349T	D450V
I10	F	Sanger, *PMR1* PCR	A557G	D186G
I10	G	Sanger, *PMR1* PCR	G533C	R178T
I10	I	Sanger, *PMR1* PCR	+ 2(AA) at bp 658	frameshift, amino acid 220
I10	J	Sanger, *PMR1* PCR	A631G	K211E
I10	K	Sanger, *PMR1* PCR	A1981C	K661Q
I10	L	Sanger, *PMR1* PCR	C1508T	A503V
I10	M	Sanger, *PMR1* PCR	G1051C	A351P
I10	Q	Sanger, *PMR1* PCR	none	n/a
I10	O	Sanger, *PMR1* PCR	none	n/a
I16	F	Sanger, *PMR1* PCR	A557G	D186G
I16	H	WGS	C554T	A185V
I16	D	WGS	G3T	start codon disruption
I16	B	Sanger, *PMR1* PCR	T412C	S138P
I16	I	Sanger, *PMR1* PCR	+ 2(AA) at bp 658	frameshift, amino acid 220
I16	N	Sanger, *PMR1* PCR	T2062G	L688V
I16	O	Sanger, *PMR1* PCR	none	n/a
I16	P	Sanger, *PMR1* PCR	G2031T	M677I

Clones from the indicated compatible (C) and incompatible (I) lines at Transfer 10 or 16 were analyzed by DNA sequencing. WGS indicates whole genome sequencing that was confirmed by Sanger sequencing the PCR-amplified *PMR1* locus. “Sanger, *PMR1* PCR” indicates that the *PMR1* locus was amplified by PCR and sequenced by the Sanger method. *pmr1* mutations are indicated by the wild-type sequence, followed by base pair or amino acid position, and then the mutant sequence. n/a, not applicable. *T to A substitution 220 bp upstream of the *PMR1* start codon.

The following observations suggested that *pmr1* mutations conferred strong adaptive advantages earlier in incompatible populations due to a higher mutation supply. 1. Almost all of the evolved incompatible clones isolated from evolved lines that completed 10 (twelve of fourteen) or 16 transfers (seven of eight) contained *pmr1* mutations. 2. Only two of ten such clones from compatible lines after Transfer 10 contained a *pmr1* mutation. 3. For compatible lines at Transfer 16, six of six such clones contained *pmr1* mutations (Table 3).

We also sequenced clones isolated from the same line, but at different transfers. For two compatible lines (lines A, N) and three incompatible lines (B, F, I) the same mutation was identified in clones isolated after Transfers 10 and 16 (Table 3). However, for an incompatible line D two different *pmr1* mutations were identified in clones isolated after Transfer 10 (*pmr1-T2213C*) and 16 (*pmr1-G3T*). Whole genome analysis of these clones showed that two indel mutations were present in both Transfer 10 and 16, suggesting that these mutations reached fixation prior to Transfer 10 (S3 Table). We then sequenced additional clones from line D incompatible line at Transfers 10 and 16. For Transfer 10, the *PMR1* gene was sequenced in nine clones; eight of these contained the T2213C mutation and one contained the wild-type sequence. For Transfer 16, all three clones that were sequenced contained the G3T mutation. Together these observations are consistent with the *T2213C* mutation being adaptive, but before it could fix the *G3T* mutation appeared in the population and swept to fixation.

Clones obtained from the compatible and incompatible evolved populations showed mutation rates that were similar to those measured in the corresponding unevolved lines (S2 Table). Thus it is not surprising that the total number of mutations detected in the evolved lines were consistent with the genotype of the line (compatible vs incompatible). On average 2.4 mutations were identified per compatible line vs. 8.0 mutations per incompatible line (p < 0.0002). Interestingly, the vast majority of indel mutations (~90%) detected in this study were in homopolymeric runs, with five times as many indels detected per line in incompatible compared to compatible lines. The latter comparison is consistent with the mutation spectra seen in *mlh1* ^*ts*^ strains grown at the non-permissive temperature [3,6].

### Gene replacement analysis shows that *pmr1* mutations identified in compatible and incompatible lines are causative for evolved salt tolerance

**Fig 5:**
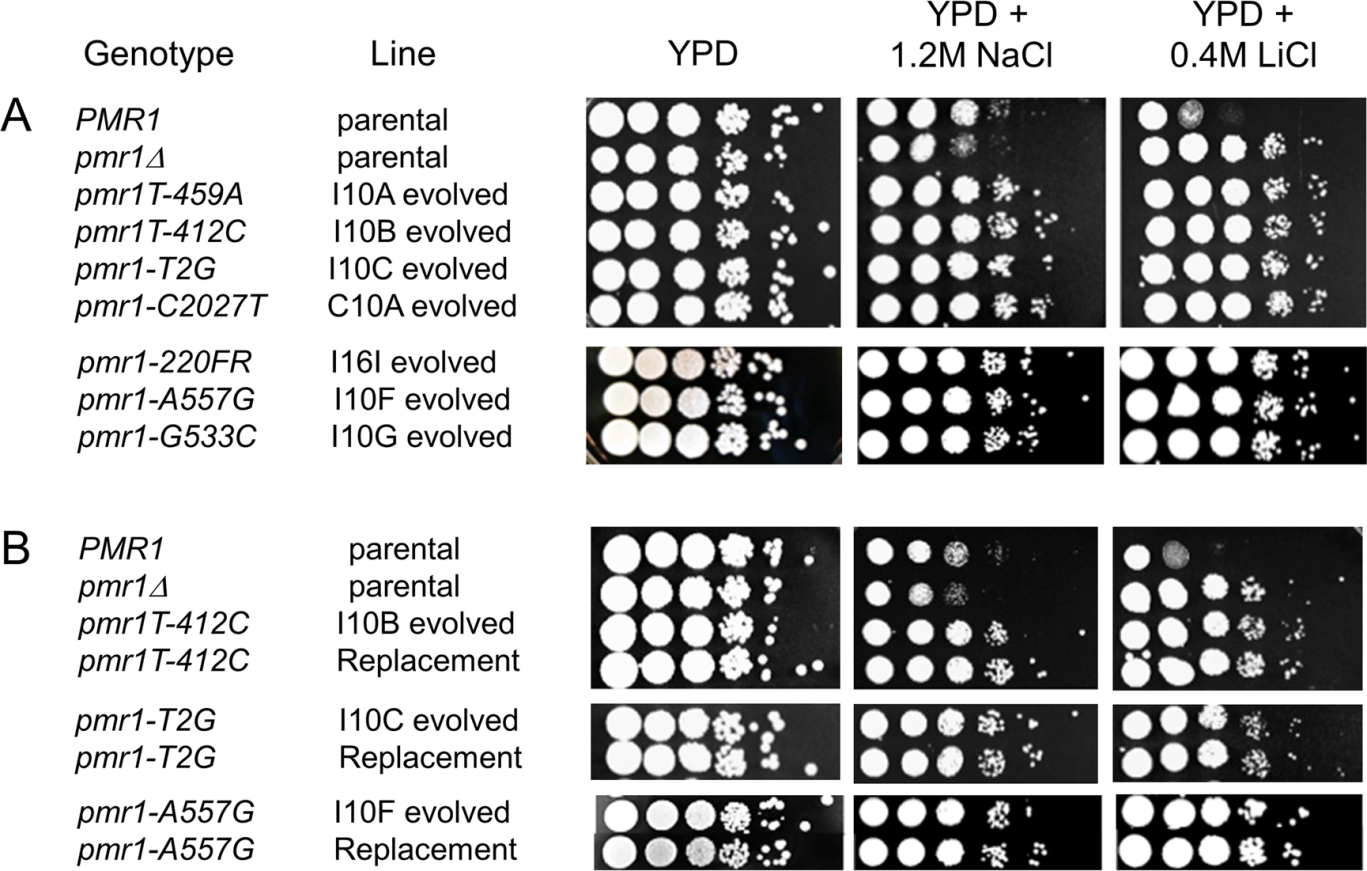
*pmr1* mutations identified in evolved lines are causative for resistance to 1.2 M NaCl and 0.4 M LiCl. In both A and B, wild type and *pmr1Δ* unevolved strains were plated in 10-fold serial dilutions onto YPD, YPD + 1.2 M NaCl, and YPD + 0.4 M LiCl plates. In panel A, representative NaCl-evolved strains (I, incompatible, C, compatible, with the Transfer indicated) bearing *pmr1* mutations are shown. In panel B, unevolved strains transformed to contain the indicated *pmr1* alleles (Replacement) are shown, with the corresponding evolved strain plated side by side.

*PMR1* encodes a P-type Ca^2+^ dependent membrane ATPase involved in protein sorting and calcium homeostasis. It is primarily localized to the Golgi membrane and is involved in transporting Mn^2+^ and Ca^2+^ into the Golgi lumen [38]. An uncharacterized *pmr1* mutation was identified by Park et al. [39] that conferred increased tolerance to NaCl. The authors proposed that such NaCl tolerance occurred because increased levels of cytosolic calcium in the *pmr1* mutant activated calcineurin, a calcium/calmodulin-dependent phosphatase. This activation increased expression of *ENA1*, a gene encoding a P-type ATPase pump that functions in sodium and lithium efflux to permit salt tolerance [39,40]. In such a model it is not surprising that *pmr1* null and hypomorph strains are also both resistant to lithium (Fig 5). However if only this pathway is involved then the *pmr1Δ* mutation should also confer NaCl tolerance. In fact *pmr1Δ* confers NaCl hypersensitivity in isogenic cell lines as well as in the S288c yeast knockout collection (Fig 5). We do not have a clear explanation for why the *pmr1* alleles identified in this study confer NaCl resistance while the *pmr1* null confers hypersensitivity. One possibility, suggested by Park et al. [39] is that factors that act in calcium homeostasis are differentially regulated in the presence or absence of full or partial-length Pmr1, and thus may differentially regulate pumps that function in sodium and lithium efflux.

To further characterize the mutations in *PMR1* and their phenotype related to salt resistance, we replaced the wild type *PMR1* gene in unevolved strains with constructs containing *pmr1* alleles identified in our studies (Fig 5). All assessed alleles (*pmr1-T412C*, *pmr1-T2G*, *pmr1-A557G*, *pmr1-C554T)* conferred NaCl^r^ and LiCl^r^ phenotypes in the unevolved strains that were identical to the phenotypes observed in the corresponding evolved strains (Fig 5). These results indicate that the phenotypes identified in evolved lines can be completely explained by mutations in *PMR1*, supporting the data shown in the bulk segregation experiments (Table 2).

To obtain a better mechanistic explanation for why *pmr1* point, frameshift, and initiation codon mutations, but not *pmr1Δ* conferred NaCl^r^, we transformed two reporter constructs, pKC201 and pMZ11, into wild-type, *pmr1Δ*, and *pmr1* allele strains. pKC201 is a *pmr2/ena1*::*lacZ* reporter plasmid used to measure expression levels of the Pmr2/Ena1 ion pump, a major P-type ATPase required for sodium ion flux. *pmr2Δ/ena1Δ* strains are sensitive to NaCl but strains containing increased copy number or expression of this locus display increased resistance [41–43]. pMZ11 is a *UPRE*::*lacZ* reporter used to monitor the unfolded protein response, a signaling pathway that improves endoplasmic reticulum (ER) function during ER stress [44].

As shown in S5 Fig, *pmr1Δ* and evolved *pmr1* strains each displayed constitutive expression of Pmr2/Ena1 at levels that were higher in the absence of NaCl than seen in wild-type. Using the pMZ11 reporter, we found that *pmr1* and *pmr1Δ* strains displayed similar phenotypes with respect to the unfolded protein response (S6 Fig). Together these results suggest that *ENA1* overexpression or the induction of the unfolded protein response cannot explain the different NaCl^r^ phenotypes seen in *pmr1* and *pmr1Δ* strains. At present we favor the idea that factors acting in calcium homeostasis are differentially regulated in the presence or absence of regulatory sequences during translation of Pmr1 or in the Pmr1 polypeptide, and may differentially regulate pumps that function in sodium and lithium efflux.

It is important to note that not all clones that showed salt resistance contained mutations in *PMR1*. In fact most NaCl^r^ clones obtained from compatible lines that had undergone 10 transfers did not contain *pmr1* mutations (S3 Table; S4 Fig). Whole genome sequencing identified mutations in other candidate genes that may be causative. For example, clone C10B isolated from the compatible line at transfer 10 (C10B) contains a mutation in *CNB1*. Cnb1 is a regulatory subunit of calcineurin that is linked to stress responses ([45]; see below). While *cnb1* null mutants show sensitivity to NaCl, mutations in *CNB1* were previously identified in lines evolved in NaCl [46]. A clone isolated from a compatible line that had completed 10 transfers (C10C) contained a mutation in *GCN2* and a clone isolated from a compatible line that had completed 16 transfers (C16B) contained a mutation in *PTK1*. Gcn2 is a protein kinase that phosphorylates the alpha-subunit of translation initiation factor eIF2 in response to starvation and Ptk1 is a putative kinase that is involved in polyamine transport [47,48]. Gene replacement approaches will be required to test whether these or other mutations identified in these clones are causative.

## Discussion

In this study we showed that *S*. *cerevisiae* populations bearing an incompatible Mlh1-Pms1 combination display an adaptive advantage when challenged to adapt to a high salt growth media. The advantage, which was rapid, but transient, was due to the greater mutation supply in incompatible populations. While *pmr1* mutations were first seen at high frequency in incompatible lines, they were eventually detected at high frequency in compatible lines. The initial fitness advantage of incompatible lines in which *pmr1* mutations had occurred no longer existed when these lines were later competed against compatible lines in which *pmr1* mutations had subsequently also arisen. Taken together, these observations suggested that the two loci MMR incompatibility genotype (*MLH1*, *PMS1*) generated by recombination among naturally occurring variants at these genes accelerated the appearance of highly beneficial mutations within our yeast populations.

Why did incompatibility accelerate adaptation? The most likely explanation is that the moderate mutator phenotype seen in incompatible strains resulted in a higher rate of mutation (including those that were neutral, deleterious, and beneficial), with strains bearing beneficial mutations selected in our high salt stress condition. Such a model fits observations seen for bacterial populations maintained in stress or selection conditions; in these experiments bacteria displaying high mutation rates were identified at high frequency (e.g. [49]). In one such study *E*. *coli* populations displaying high mutation rates, primarily due to MMR defects, showed short-term fitness advantages that were not sustainable [19]. The simplest explanation is that the lack of a long-term fitness advantage was due to the accumulation of deleterious mutations elsewhere in the genomes containing the beneficial mutations [19,50–52]. As indicated in the Introduction, bacterial populations appear to overcome long-term fitness costs associated with high mutation rates by reacquiring functional MMR genes and thus normal mutation rates through horizontal gene transfer [14,19].

Several groups have examined whether a mutator phenotype in eukaryotes confers a fitness advantage when adapting to a stress environment (e.g. [21,37]). Some of the best-known examples involve cancer cells that display mutator phenotypes and/or high rates of genome instability [53]. Such work has suggested that over time mutator populations lose their initial advantage due to fitness costs and clonal interference (e.g. [37]). Fitness costs associated with high mutation rates can occur rapidly; for example, Ma et al. [6] showed that, following 160 generations of growth in non-permissive conditions, a diploid *mlh1* ^*ts*^ yeast strain accumulated 92 heterozygous mutations, including five in essential genes, and that these mutations account for the poor spore viability (3%) seen in the strain. In addition even a moderate mutator can quickly display a fitness defect. Relevant to this study, Heck et al. [22] showed that the *S288c MLH1-SK1 PMS1* incompatible genotype conferred a subtle but significant fitness cost, as measured by decreased spore viability, in diploids grown in rich media for 160 generations. These observations suggest that an adaptive advantage seen in a mutator population is not sustainable. Furthermore, horizontal gene transfer is very rare in yeast [54], indicating that MMR is unlikely to be recovered through such a mechanism.

In our studies the incompatibility seen in *S288c MLH1-SK1 PMS1* strains provided an initial adaptive advantage prior to observing a detectable fitness cost. Such an advantage was likely due to incompatible strains displaying a modest mutator phenotype in conjunction with relatively large population sizes of the yeast undergoing serial transfer. How can adapted eukaryotic strains that are mutators escape long-term fitness costs? Sequencing analysis of a 32 kb genomic region provided evidence for recombination between SK1 and S288c strain groups [22]. This observation suggests that an incompatibility could be generated through mating and that adapted incompatible populations can mate back to other strains available in the environment to regain a compatible MMR combination, thus avoiding long-term fitness costs. Several factors are thought to contribute to the likelihood of such a scenario: 1. The frequently of meiotic cycles in wild populations. 2. The number of clonal generations experienced between an outcross. 3. The effects of post-zygotic barriers on the formation of viable progeny. 4. The effect of a stress condition on mating and meiotic cycles.

The ratio between mitotic and meiotic cycles in wild populations of *S*. *cerevisiae* is not known, although in *S*. *paradoxus*, population genetics approaches have shown that this organism undergoes a sexual cycle approximately once every 1,000 asexual cycles [55]. Magwene et al. [56] used a molecular clock analysis of genomic sequences between yeast strains to estimate the number of clonal generations that two strains would have experienced prior to outcrossing. Their estimates, in conjunction with recombination frequency estimates performed by Ruderfer et al. [57], suggested one outcrossing event per 12,500 to 62,500 generations. Importantly, random mating between spores in natural strains can occur at high rates in the laboratory, and outbreeding was shown to be elevated when spores from different strains were passaged through the fruit fly gut [58,59]. Therefore mating behaviors are likely to be affected by yeast lifestyle conditions that include selection/stress conditions, MMR defects, and population size. Thus it is not difficult to imagine outcrossing restoring MMR compatibility in a large population subjected to strong selection.

### Mutations in *PMR1* confer a striking adaptive advantage to NaCl tolerance

The majority of mutations that conferred salt tolerance mapped to the *PMR1* locus (Table 2 and Fig 5). Previously Anderson et al. [42] identified mutations in other loci that are linked to NaCl tolerance including *PMA1*, which encodes a proton efflux pump, *ENA1*, which encodes a sodium efflux pump, and *CYC8*, which encodes a global transcriptional repressor that regulates ENA1 activity [42]. Possible reasons for why different loci were targeted in the two studies include: 1. The strains used in the two studies were not identical and were likely to have different background mutations. 2. We imposed a stronger selection for NaCl tolerance (1.2 M) than Anderson et al. (1.0 M) [42]. 3. We screened for adaptive advantages at earlier generations (70–100) than Anderson et al. (100–500) [42]. This is of interest because of recent observations made by Lang et al. [60], who studied the appearance of beneficial sterility mutations in haploid *S*. *cerevisiae*. In their system they estimated that roughly 100 generations of adaptation were required to generate a threshold level of genetic diversity upon which beneficial mutations could be selected. Thus different target genes might be identified depending on when adaptation is measured. 4. The effective population size per transfer is different in the two studies; we used a 10-fold higher number of cells than Anderson et al. [42]. Such a difference would likely alter the frequency and likelihood that mutations in any one locus would emerge. It is important to note that despite the differences in genes identified between the two studies there is a nice commonality in that NaCl^r^ in both studies is likely to involve altered regulation of the Ena1 efflux pump (S5 Fig).

### Negative epistasis in MMR genes as a possible adaptation strategy

Epistatic effects involving interacting alleles have been detected for specific fitness measurements between individuals within a population (e.g. [61]). One of the best demonstrations of such effects in yeast was obtained by Brem et al. [62], who crossed two strains of baker’s yeast and then searched for genetic interactions by measuring the levels of all transcripts in a large number of spore progeny. In their analysis they identified statistically significant interactions between locus pairs for 225 transcripts. Based on a population survey of *MLH1* and *PMS1* alleles, we argued previously that the incompatibility that was identified between MMR genes is similar to epistatic interactions seen in hybrids formed from established or incipient species ([28,29]; see examples in [22]). Support for such an idea is based on the fact that mild reproductive barriers have already been shown to exist between some *S*. *cerevisiae* strains [22], and the MMR machinery has been shown to contribute to reproductive isolation when *S*. *cerevisiae* strains with sequence divergence are mated [63,64]. The experiments presented in this paper provide an interesting twist to this idea because the incompatibility involving *MLH1* and *PMS1* might also provide opportunities for adaptive evolution by moderately increasing mutation rates.

## Materials and Methods

### Media, plasmids and strains

Yeast strains, all isogenic to the FY/S288c background, were grown in YPD (yeast extract, peptone, dextrose), YPD + 1.2 M sodium chloride (NaCl), or YPD + 0.4 M lithium chloride (LiCl) (S1 Table; [65,66]). DNA fragments containing S288c or SK1 derived *MLH1* and *PMS1* genes *(MLH1*:*KANMX*, *MLH1*::*NATMX*, and *PMS1*::*HIS3)* were introduced into S288c-background strains by gene replacement (S1 Table; [22,67]). S288c derived *pmr1*::*URA3* alleles (*URA3* is located 500 bp upstream of *PMR1*) were also introduced into S288c background strains by gene replacement (S1 Table, pEAA602-606 digested with *Not*I and *Xho*I). Integrations were confirmed by PCR amplification of yeast chromosomal DNA, prepared as described by Holm et al. [68] using primers located outside of the ends of the DNA fragments used for integration. Allele integrations were confirmed by sequencing the relevant PCR products using the Sanger method. The sequences of the oligonucleotides used to perform PCR are available upon request. pMZ11 (*UPRE*::*lacZ*, *ARS*-*CEN*, *TRP1*, reporter to measure the unfolded protein response) and pKC201 (*pmr2*::*lacZ*, *2μ*, *URA3* reporter to measure *PMR2* expression) were generously provided by Jeff Brodsky and Kyle Cunningham, respectively.

### Adaptive evolution assays

Single colonies of *Saccharomyces cerevisiae* were inoculated into 6 ml of YPD and grown for 24 hrs at 30°C in 20 ml glass tubes in a New Brunswick G25 shaker run at 250 RPM. Approximately 2 x 10^7^ of each culture were then transferred into fresh 6 ml YPD or YPD + 1.2 M NaCl (to achieve an initial OD_600_ of 0.1, Shimadzu UV-1201 spectrophotometer) and then grown for 24 hrs. This procedure was repeated for up to 20 transfers. The number cell generations completed per transfer was determined using the equation log_2_ (N_t_/N_o_), where N_o_ = total cell count at 0 hrs and N_t_ = total cell count at 24 hrs post transfer. A Wilcoxon sign-ranked test was used to compare growth of independent cultures [69].

### Competition assays

Incompatible and compatible *MLH1-PMS1* strains were created in which the *MLH1* gene was marked with *KANMX* or *NATMX* (S1 Table). These markers were shown previously to not affect fitness [70,71]. After 7, 10, and 16 transfers in YPD or YPD + 1.2 M NaCl (approximately 50, 70 and 110 generations respectively), incompatible and compatible populations were mixed at a 1:1 ratio (1 x 10^7^ cells each inoculated into 5 ml YPD or YPD + 1.2 M NaCl) and grown for an additional 24 hours. The ratio of incompatible and compatible populations was assessed by replica plating YPD plates containing ~ 200 yeast colonies (plated prior to, or after 24 hrs of growth) onto YPD-G418 and YPD-nourseothricin plates [70].

Fitness values (Table 1) were separately determined after competition experiments in which evolved cultures were randomly mixed at a 1:1 ratio and grown for an additional 24 hours (estimated to be 7 generations) in YPD or YPD + 1.2 M NaCl. Fitness (*w*) [34,35] of the incompatible cells relative to the compatible cells was calculated as *w* = ((*p*_*t*_ */q*_*t*_)/(*p*_*o*_ */q*_*o*_))^1/t^, where *p*_*o*_ and *q*_*o*_ are the number of incompatible and compatible cells, respectively at 0 hrs and *p*_*t*_ and *q*_*t*_ are the number of incompatible and compatible cells, respectively, at 24 hrs, with t = 7 generations of growth. Fitness differences were analyzed for significance using one-way ANOVA [36].

### *lys2-A*_*14*_ reversion assays

*lys2-A*_*14*_ strains (S1 Table; (A)_14_ inserted into the *LYS2* gene) were analyzed for reversion to Lys^+^ as described previously [27,72]. All strains were inoculated in YPD overnight and plated onto LYS drop out and synthetic complete plates. The 95% confidence intervals were determined as described by Dixon and Massey [73]. Pair-wise Kruskal–Wallis tests were performed between each pair of incompatible and compatible strains to determine the significance of the differences in median reversion rates.

### Bulk segregation analysis

Individual NaCl^r^ clones isolated from incompatible and compatible strains grown for 10 transfers were phenotyped and then crossed to isogenic, unevolved strains. The resulting diploids were first struck onto YPD + 1.2 M NaCl plates to determine if the NaCl^r^ phenotype observed in the evolved haploid strain was dominant or recessive. The diploids were then sporulated using either liquid or solid media containing 1% potassium acetate. Tetrads were dissected and spores clones germinated on YPD were struck onto YPD + 1.2 M NaCl to assess NaCl resistance. The resulting NaCl^r^ and NaCl^s^ spore clones, at least 18 of each, were pooled in equal cell amounts to create resistant and sensitive bulk pools that were subjected to whole genome sequencing.

### Whole genome sequencing

Parental, NaCl^r^ evolved compatible and incompatible clones, and the bulk pools described above, were grown in 8 ml cultures. Chromosomal DNA was isolated using Affymetrix Prep-Ease kit and quantified using the Qubit dsDNA HS Assay Kit (Life Technologies). This DNA was then barcoded using Illumina Nextera XT. High throughput sequencing of chromosomal DNA was performed on an Illumina HiSeq 2500 at the Cornell Biotechnology Resource Center.

GATK was used as a platform-independent Java framework. The core system uses the standard sequence alignment program BWA against a reference sequence to create SAM format files [74]. We used the S288c reference sequence in this study (SGD: http://www.yeastgenome.org/) because our strains are isogenic to this background. All differences between our starting unevolved strain and the reference were subtracted. A binary alignment version of the SAM format, called binary alignment/map (BAM), was then compressed and indexed using picard (http://picard.sourceforge.net). Finally, BAM files were analyzed by GATK to optimize the genotyping analysis (http://www.broadinstitute.org/gsa/wiki/index.php/The_Genome_Analysis_Toolkit). SNPs with quality scores of less than 75 were removed from the analysis.

Uniprot was used to predict the topology of Pmr1 (http://www.uniprot.org/).

### Beta-galactosidase assays

pKC201 (*pmr2*::*lacZ*, *2μ*, *URA3;* [41]) was transformed into evolved strains to determine if mutations in *PMR1* activated *ENA1/PMR2* expression. Transformants were grown overnight in uracil dropout media in the presence or absence of 1.2 M NaCl and then analyzed in liquid assays for beta-galactosidase activity (permeabilized yeast cell assay, [51]). pMZ11 (*UPRE*::*lacZ*, *ARS-CEN*, *TRP1;* [44]) was transformed into evolved strains to determine if mutations in *PMR1* activated the unfolded protein response pathway. Transformants were grown for the indicated times in tryptophan dropout media with or without 1.2 M NaCl and then analyzed in liquid assays for beta-galactosidase activity. DTT was included at 5 mM to serve as a positive control for the unfolded protein response.

## Supporting Information

S1 Fig Unevolved compatible and incompatible strains display similar fitness. Unevolved, compatible (*kMLH1-kPMS1*, EAY3242) and incompatible (*cMLH1-kPMS1*, EAY3236) strains were grown to saturation in YPD and then diluted to an initial OD_600_ of 0.1 in YPD (A) or YPD + 1.2 M NaCl (B). Independent cultures were then monitored for growth at 30°C for up to 12 hrs. A representative experiment involving three replicates for each genotype is shown. Mean OD_600_, +/- standard deviation, is presented for each time point.
(TIFF)

S2 Fig Representative competition experiments showing that incompatible strains display a transient fitness advantage in adapting to NaCl. Independent cultures of compatible (*kMLH1-kPMS1*, EAY3242) and incompatible (*cMLH1-kPMS1*, EAY3236) strains were subjected to 0 (Panel A, initial strains) 7 (B), 10 (C), and 16 (D) transfers (2 x 10^7^ cells per transfer) in YPD (left) or YPD + 1.2 M NaCl (right). Left panels: incompatible and compatible cultures transferred in YPD were randomly mixed at a 1:1 ratio and grown for an additional 24 hours in YPD. Right panels: Incompatible and compatible cultures transferred in YPD + 1.2 M NaCl were randomly mixed at a 1:1 ratio and grown for an additional 24 hours in YPD + 1.2 M NaCl. In both sets of experiments, the ratio of incompatible to compatible populations is presented prior to and after 24 hrs of growth. See Materials and Methods for details.
(TIFF)

S3 Fig Incompatible fitness advantage is not observed when the amount of cells transferred is reduced 10-fold. Independent cultures of compatible (*kMLH1-kPMS1*, EAY3242) and incompatible (*cMLH1-kPMS1*, EAY3236) strains were grown for up to 16 transfers (~ 2 x 10^6^ cells per transfer) in YPD (unevolved) or YPD + 1.2 M NaCl (evolved). In A, data are shown in which cultures after Transfer 10 were diluted to an OD_600_ of 0.1 in YPD + 1.2 M NaCl and monitored for growth for 12 hrs. A representative experiment involving three replicates for each genotype is shown. Mean OD_600_, +/- standard deviation, is presented for each time point.
(TIFF)

S4 Fig Growth of compatible lines on salt media. NaCl^r^ clones (Table 3) obtained from Transfer 10 compatible lines were plated in 10-fold serial dilutions onto YPD, YPD + 1.2 M NaCl and YPD + 0.4 M LiCl plates.
(TIFF)

S5 Fig *pmr1* mutant strains constitutively induce expression of *ENA1/PMR2*. Wild-type, *pmr1Δ*, and NaCl^r^ strains bearing the indicated *pmr1* mutations were transformed with the *ena1*::*LACZ* reporter pKC201 to measure Ena1 expression. Transformants were analyzed in the presence or absence of NaCl for beta-galactosidase activity as described in the Materials and Methods. The standard deviation of 4–8 independent measurements is presented.
(TIFF)

S6 Fig *pmr1* mutant strains do not appear to be induced for the unfolded protein response. Wild-type, *pmr1Δ*, and NaCl^r^ strains bearing the indicated *pmr1* mutations were transformed with the *UPRE*::*LACZ* reporter pMZ11 to measure the unfolded protein response. Transformants were analyzed for beta-galactosidase activity as described in the Materials and Methods. DTT was included at a final concentration of 5 mM and NaCl was added at a final concentration of 1.2 M for the number of hours indicated. The standard deviation of 2–7 independent measurements is presented.
(TIFF)

S1 Table Strains and plasmids used in this study. S288c derived genes are referred to as “c” and SK1 derived genes as “k.”
(DOCX)

S2 Table Mutation rates in unevolved and evolved compatible and incompatible *MLH1-PMS1* strains. The *lys2*:*InsE-A*_*14*_ strains EAY3234 (*cMLH1-cPMS1*, *compatible*, *c-c*), EAY3225 (*kMLH1-cPMS1*, *compatible*, *k-c*), EAY3246 (*kMLH1-kPMS1*, *compatible*, *k-k*), EAY3235 (*cMLH1-kPMS1*, *incompatible*, *c-k*), and evolved NaCl-resistant clones from these strains obtained from Transfer 10 were examined for reversion to Lys^+^. n, the number of independent cultures tested. Median mutation rates are presented with 95% confidence intervals, and relative mutation rates compared to the wild type strain are shown. *Data for *mlh1Δ* (EAY1366) were obtained from Wanat et al. [75].
(DOCX)

S3 Table Whole genome sequencing of mutations in single clones isolated from evolved strains. SNP and Indel mutations were identified by whole genome sequencing of one individual clone purified from each of ten independently evolved populations (Materials and Methods). The individual clones (A, B, C, etc.) are indicated as being from incompatible (I) or compatible (C) strains evolved for 10 or 16 transfers. nc indicates a SNP or indel was detected in a non-coding region.
(DOCX)
