# Supplementary figures and images for: A Genetic Incompatibility Accelerates Adaptation in Yeast
Source: PLoS Genet. 2015 Jul 31;11(7):e1005407. doi: 10.1371/journal.pgen.1005407 (PMC4521705; doi:10.1371/journal.pgen.1005407)

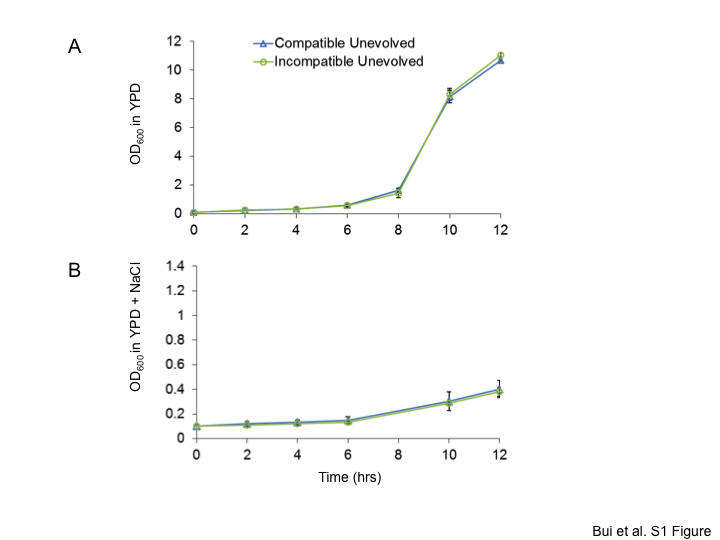

Supplement: S1 Fig — Unevolved, compatible (kMLH1-kPMS1, EAY3242) and incompatible (cMLH1-kPMS1, EAY3236) strains were grown to saturation in YPD and then diluted to an initial OD600 of 0.1 in YPD (A) or YPD + 1.2 M NaCl (B). Independent cultures were then monitored for growth at 30°C for up to 12 hrs. A representative experiment involving three replicates for each genotype is shown. Mean OD600, +/- standard deviation, is presented for each time point. (TIFF) [file pgen.1005407.s001.tiff]

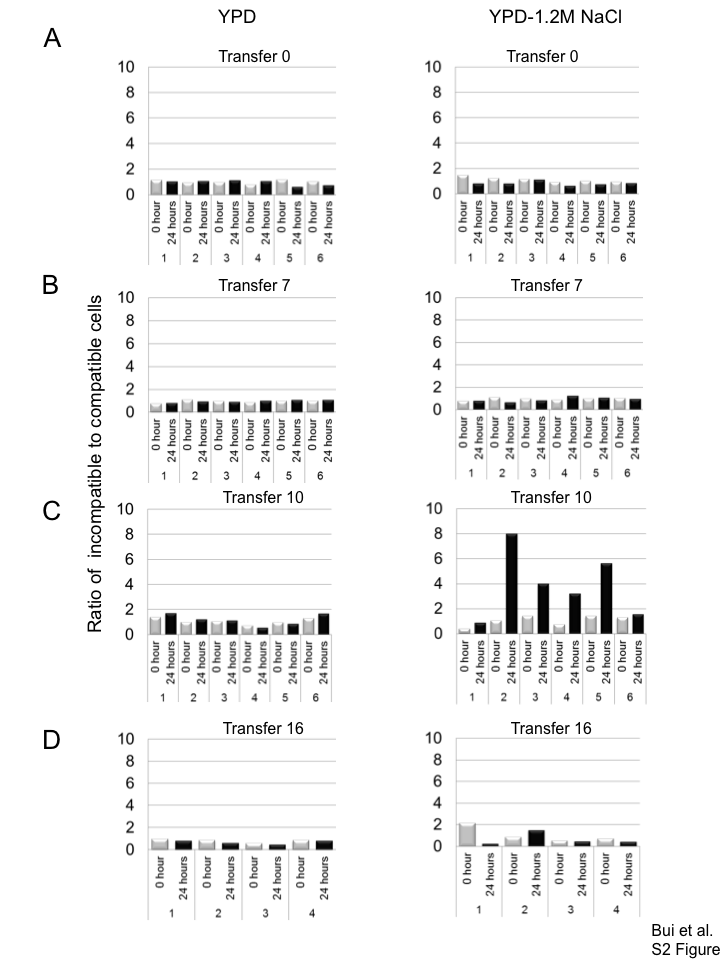

Supplement: S2 Fig — Independent cultures of compatible (kMLH1-kPMS1, EAY3242) and incompatible (cMLH1-kPMS1, EAY3236) strains were subjected to 0 (Panel A, initial strains) 7 (B), 10 (C), and 16 (D) transfers (2 x 10^7^ cells per transfer) in YPD (left) or YPD + 1.2 M NaCl (right). Left panels: incompatible and compatible cultures transferred in YPD were randomly mixed at a 1:1 ratio and grown for an additional 24 hours in YPD. Right panels: Incompatible and compatible cultures transferred in YPD + 1.2 M NaCl were randomly mixed at a 1:1 ratio and grown for an additional 24 hours in YPD + 1.2 M NaCl. In both sets of experiments, the ratio of incompatible to compatible populations is presented prior to and after 24 hrs of growth. See Materials and Methods for details. (TIFF) [file pgen.1005407.s002.tiff]

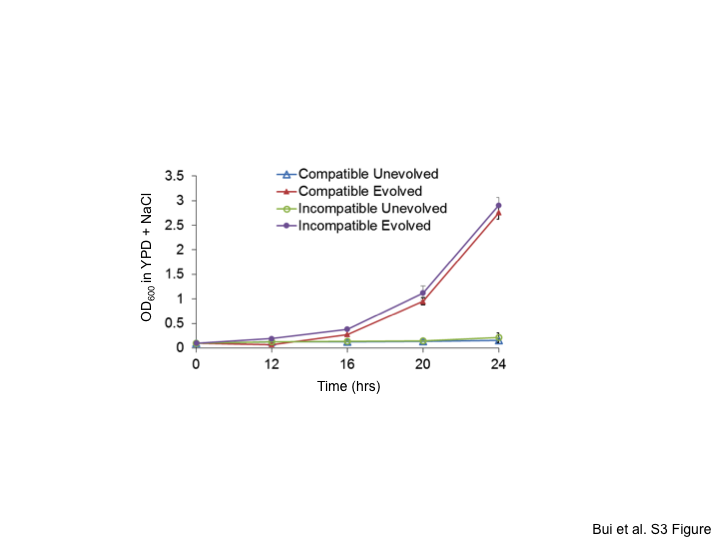

Supplement: S3 Fig — Independent cultures of compatible (kMLH1-kPMS1, EAY3242) and incompatible (cMLH1-kPMS1, EAY3236) strains were grown for up to 16 transfers (~ 2 x 10^6^ cells per transfer) in YPD (unevolved) or YPD + 1.2 M NaCl (evolved). In A, data are shown in which cultures after Transfer 10 were diluted to an OD600 of 0.1 in YPD + 1.2 M NaCl and monitored for growth for 12 hrs. A representative experiment involving three replicates for each genotype is shown. Mean OD600, +/- standard deviation, is presented for each time point. (TIFF) [file pgen.1005407.s003.tiff]

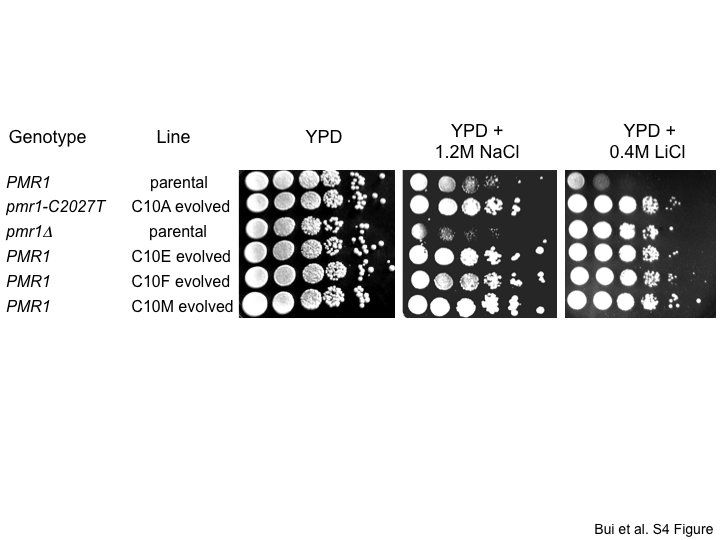

Supplement: S4 Fig — NaClr clones (Table 3) obtained from Transfer 10 compatible lines were plated in 10-fold serial dilutions onto YPD, YPD + 1.2 M NaCl and YPD + 0.4 M LiCl plates. (TIFF) [file pgen.1005407.s004.tiff]

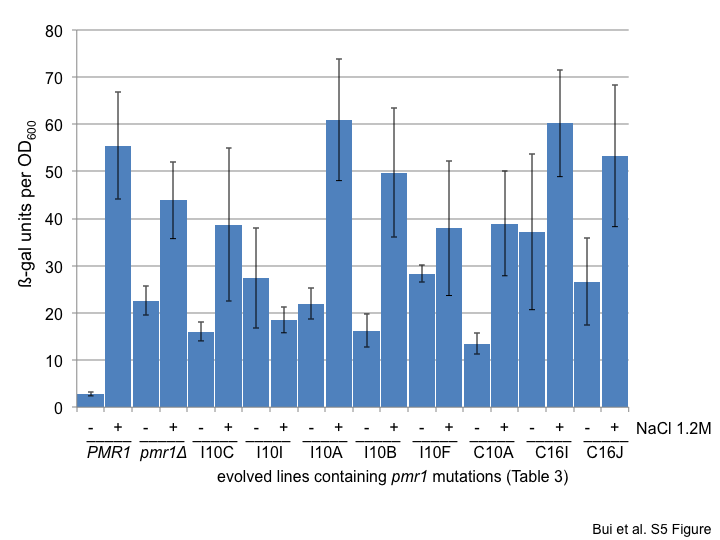

Supplement: S5 Fig — Wild-type, pmr1Δ, and NaClr strains bearing the indicated pmr1 mutations were transformed with the ena1::LACZ reporter pKC201 to measure Ena1 expression. Transformants were analyzed in the presence or absence of NaCl for beta-galactosidase activity as described in the Materials and Methods. The standard deviation of 4–8 independent measurements is presented. (TIFF) [file pgen.1005407.s005.tiff]

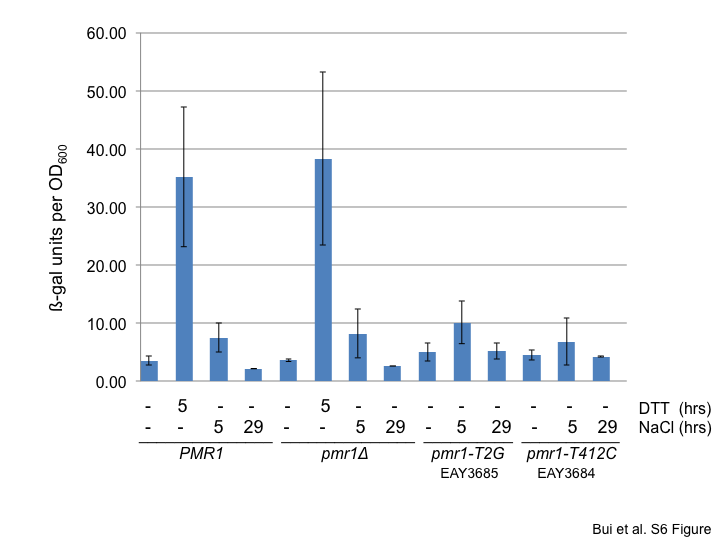

Supplement: S6 Fig — Wild-type, pmr1Δ, and NaClr strains bearing the indicated pmr1 mutations were transformed with the UPRE::LACZ reporter pMZ11 to measure the unfolded protein response. Transformants were analyzed for beta-galactosidase activity as described in the Materials and Methods. DTT was included at a final concentration of 5 mM and NaCl was added at a final concentration of 1.2 M for the number of hours indicated. The standard deviation of 2–7 independent measurements is presented. (TIFF) [file pgen.1005407.s006.tiff]
