# Supplementary material for: A Genetic Incompatibility Accelerates Adaptation in Yeast
Source: PLoS Genet. 2015 Jul 31;11(7):e1005407. doi: 10.1371/journal.pgen.1005407 (PMC4521705; doi:10.1371/journal.pgen.1005407)
Supplement: S1 Table — S288c derived genes are referred to as “c” and SK1 derived genes as “k.” (DOCX) [file pgen.1005407.s007.docx]

| **S1 Table. Strains and plasmids used in this study.** | | |
| --- | --- | --- |
|  | |  |
| Strains |  |  |
| FY23 | *MATa, ura3-52, leu2∆1, trp1∆63* |  |
| FY86 | *MATalpha, ura3-52, leu2∆1, his3∆200* |  |
| EAY1366 | *MATa, leu2, ura3, trp1, his3, lys2::InsE-A14, mlh1∆::KANMX* |  |
| EAY1369 | *MATalpha, ura3-52, leu2∆1, trp1∆63, his3∆200, lys2::insE-A14, cPMS1::HIS3* |  |
| EAY1370 | *MATalpha, ura3-52, leu2∆1, trp1∆63, his3∆200, lys2::insE-A14, kPMS1::HIS3* |  |
| EAY3191 | *MATa, ura3-52, leu2∆1, trp1∆63, his3∆200, kMLH1::KANMX, kPMS1::HIS3* |  |
| EAY3193 | *MATalpha, ura3-52, leu2∆1, trp1∆63, his3∆200, kMLH1::KANMX, kPMS1::HIS3,* |  |
| EAY3197 | *MATalpha, ura3-52, leu2∆1, trp1∆63, his3∆200, cMLH1::NATMX, kPMS1::HIS3* |  |
| EAY3225 | *MATalpha, ura3-52, leu2∆1, trp1∆63, his3∆200, lys2::insE-A14, kMLH1::NATMX, cPMS1::HIS3* |  |
| EAY3230 | *MATalpha, ura3-52, leu2∆1, trp1∆63, his3∆200, kMLH1::NATMX, cPMS1::HIS3* |  |
| EAY3233 | *MATalpha, ura3-52, leu2∆1, trp1∆63, his3∆200, cMLH1-KANMX, cPMS1::HIS3* |  |
| EAY3234 | *MATa, ura3-52,leu2∆1, trp1∆63, his3∆200, lys2::insE-A14, cMLH1-KANMX, cPMS1::HIS3* |  |
| EAY3235 | *MATa, ura3-52, leu2∆1, trp1∆63, his3∆200, lys2::insE-A14, cMLH1::KANMX, kPMS1::HIS3* |  |
| EAY3236 | *MATalpha, ura3-52,leu2∆1, trp1∆63, his3∆200, cMLH1::KANMX, kPMS1::HIS3* |  |
| EAY3239 | *MATalpha, ura3-52, leu2∆1, trp1∆63, his3∆200, kMLH1::NATMX, kPMS1::HIS3* |  |
| EAY3241 | *MATa, ura3-52, leu2∆1, trp1∆63, his3∆200, kMLH1::NATMX, kPMS1::HIS3* |  |
| EAY3242 | *MATalpha ura3-52, leu2∆1, trp1∆63, his3∆200, kMLH1::NATMX, kPMS1::HIS3* |  |
| EAY3246 | *MATa, ura3-52,leu2∆1, trp1∆63, his3∆200, lys2::insE-A14, kMLH1::NATMX, kPMS1::HIS3* |  |
| EAY3247 | *MAT alpha, ura3-52, leu2∆1, trp1∆63, his3∆200, lys2::insE-A14, kMLH1::NATMX kPMS1::HIS3* |  |
| EAY3684 | *EAY3242, pmr1-T412C::URA3* |  |
| EAY3685 | *EAY3242, pmr1-T2G::URA3* |  |
| EAY3686 | *EAY3242, pmr1-A557G::URA3* |  |
| EAY3687 | *EAY3242, pmr1-C554T::URA3* |  |
| EAY3688 | *EAY3242, PMR1::URA3* |  |
|  |  |  |
| Plasmids | |  |
| pRS416 | *ARS-CEN, URA3* |  |
| pEAA588 | *ARS-CEN, URA3, PMR1* |  |
| pEAA589 | *ARS-CEN, URA3, pmr1-C2027T* |  |
| pEAA590 | *ARS-CEN, URA3, pmr1-T459A* |  |
| pEAA591 | *ARS-CEN, URA3, pmr1-T412C* |  |
| pEAA592 | *ARS-CEN, URA3, pmr1-T2G* |  |
| pEAA594 | *ARS-CEN, URA3, pmr1-G2031T* |  |
| pEAA595 | *ARS-CEN, URA3, pmr1-A557G* |  |
| pEAA600 | *ARS-CEN, URA3, pmr1-658AAinsertion* |  |
| pEAA601 | *ARS-CEN, URA3, pmr1-A778C* |  |
| pEAA602 | *ARS-CEN, URA3, PMR1::URA3* |  |
| pEAA603 | *ARS-CEN, URA3, pmr1-T412C::URA3* |  |
| pEAA604 | *ARS-CEN, URA3, pmr1-T2G::URA3* |  |
| pEAA605 | *ARS-CEN, URA3, pmr1-A557G::URA3* |  |
| pEAA606 | *ARS-CEN, URA3, pmr1-C554T::URA3* |  |
| pMZ11 | *ARS-CEN, TRP1, UPRE::lacZ* |  |
| pKC201 | *2 micron, URA3, pmr2A::lacZ* |  |
|  |  |  |
| S288c derived genes are referred to as “c” and SK1 derived genes as “k” | |  |
