# Supplementary material for: A Genetic Incompatibility Accelerates Adaptation in Yeast
Source: PLoS Genet. 2015 Jul 31;11(7):e1005407. doi: 10.1371/journal.pgen.1005407 (PMC4521705; doi:10.1371/journal.pgen.1005407)
Supplement: S2 Table — The lys2:InsE-A 14 strains EAY3234 (cMLH1-cPMS1, compatible, c-c), EAY3225 (kMLH1-cPMS1, compatible, k-c), EAY3246 (kMLH1-kPMS1, compatible, k-k), EAY3235 (cMLH1-kPMS1, incompatible, c-k), and evolved NaCl-resistant clones from these strains obtained from Transfer 10 were examined for reversion to Lys+. n, the number of independent cultures tested. Median mutation rates are presented with 95% confidence intervals, and relative mutation rates compared to the wild type strain are shown. *Data for mlh1Δ (EAY1366) were obtained from Wanat et al. [75]. (DOCX) [file pgen.1005407.s008.docx]

**S2 Table. Mutation rates in in unevolved and evolved compatible and incompatible *MLH1-PMS1* strains.**

*MLH1-PMS1* genotype Lys^+^ reversion rate (10^-7^), (95% CI) Relative Rate (n)

___________________________________________________________________________________________________

Compatible (c-c) 1.55 (1.14-1.96) 1 35

Compatible (c-c), Clone 1, Transfer 10 1.58 (1.21-2.57) 1.0 19

Compatible (c-c), Clone 2, Transfer 10 2.43 (2.2-3.3) 1.7 5

Compatible (c-c), Clone 3, Transfer 10 2.8 (2.27-3.23) 1.9 10

Compatible (k-k) 11.1 (8.9-13.8) 7.2 30

Compatible (k-k), Clone 1, Transfer 10 5.67 (1.24-19.3) 3.7 10

Compatible (k-k), Clone 2, Transfer 10 11.1 (7.4-15.2) 7.7 5

Compatible (k-k), Clone 3, Transfer 10 10.6 (6.8-16.7) 7.3 5

Compatible (k-k), Clone 1, Transfer 16 15.8 (13-25.3) 10.9 15

Compatible (k-k), Clone 2, Transfer 16 10.9 (8.7-20.6) 7.5 5

Compatible (k-k), Clone 3, Transfer 16 8.4 (5.3-6.46) 5.8 10

Incompatible (c-k) 183 (162-335) 118 44

Incompatible (c-k), Clone 1, Transfer 10 89.3 (46.2-160) 57.6 20

Incompatible (c-k), Clone 2, Transfer 10 88.8 (60.0-191) 57.3 10

Incompatible (c-k), Clone 3, Transfer 10 93.8 (43.5-149) 60.5 15

Incompatible (c-k), Clone 1, Transfer 16 119.4(85.5-185) 77 15

Incompatible (c-k), Clone 2, Transfer 16 169 (119-244) 116.6 8

Incompatible (c-k), Clone 3, Transfer 16 151 (104-200) 104.1 5

Compatible (k-c) 1.07 (0.70-1.92) 0.7 15

*mlh1Δ** 31,950 (15,900-60,150) 20,600 25

____________________________________________________________________________________________________

The *lys2:InsE-A_14_* strains EAY3234 (*cMLH1-cPMS1, compatible, c-c*), EAY3225 (*kMLH1-cPMS1, compatible, k-c*), EAY3246 (*kMLH1-kPMS1, compatible, k-k*), EAY3235 (*cMLH1-kPMS1, incompatible, c-k*), and evolved NaCl-resistant clones from these strains obtained from Transfer 10 were examined for reversion to Lys^+^. n, the number of independent cultures tested. Median mutation rates are presented with 95% confidence intervals, and relative mutation rates compared to the wild type strain are shown. *Data for *mlh1Δ* (EAY1366) were obtained from Wanat et al. [75].
