# Supplementary material for: A Genetic Incompatibility Accelerates Adaptation in Yeast
Source: PLoS Genet. 2015 Jul 31;11(7):e1005407. doi: 10.1371/journal.pgen.1005407 (PMC4521705; doi:10.1371/journal.pgen.1005407)
Supplement: S3 Table — SNP and Indel mutations were identified by whole genome sequencing of one individual clone purified from each of ten independently evolved populations (Materials and Methods). The individual clones (A, B, C, etc.) are indicated as being from incompatible (I) or compatible (C) strains evolved for 10 or 16 transfers. nc indicates a SNP or indel was detected in a non-coding region. (DOCX) [file pgen.1005407.s009.docx]

| **S3 Table. Whole genome sequencing of mutations in single clones isolated from evolved strains.** | | | | | | |
| --- | --- | --- | --- | --- | --- | --- |
| evolved  clone | | SNPs, location of mutations | | Indels, location of mutations | | Average SNPs + INDELs |
| C10 | A | 1 | chrVII 188442 *PMR1* | 0 |  | 3.3 |
|  |  |  |  |  |  |  |
|  |  |  |  |  |  |  |
| C10 | B | 1 | chrXI, 83451, *CNB1* | 2 | chrIV, 966520, *RMD5* |  |
|  |  |  |  |  | chrXV, 444498, *RPL3* |  |
|  |  |  |  |  |  |  |
| C10 | C | 1 | chrIV, 1028956, *GCN2* | 1 | chrXII, 1054059, YLR455W |  |
|  |  |  |  |  |  |  |
| C16 | A | 2 | chrVI, 113096, *HXT10* | 0 |  | 5 |
|  |  |  | chrVII, 188842, *PMR1* |  |  |  |
|  |  |  |  |  |  |  |
| C16 | G | 2 | chrIV, 822440, *SCC1* | 2 | chrI, 70179, nc |  |
|  |  |  | chrVII, 190689 (221 bp upstream of *PMR1* start codon and 440 bp upstream of the *CUP2* start codon) | | chrXI, 68454, *PTK1* |  |
|  |  |  |  |  |  |  |
| I10 | A | 4 | chrVII, 190010, *PMR1* | 4 | chrI, 9052, nc, 32 bp upstream of *SEO1* start codon | 7.3 |
|  |  |  | chrVIII, 555937, *IMD2* |  | chrIII, 315517, nc |  |
|  |  |  | chrXI, 381005, *IXR1* |  | chrIV, 977331, nc |  |
|  |  |  | chrXVI, 50390, *RAD1* |  | chrXIII, 858326, IBI2 |  |
|  |  |  |  |  |  |  |
| I10 | D | 3 | chrVII, 188256, *PMR1* | 6 | chrII, 602369, nc (265 upstream of the *GDT1* start codon) |  |
|  |  |  | chrIX, 101521, *FKH1* |  | chrII, 767793, nc (187 bp upstream of the *DUG2* start codon) |  |
|  |  |  | chrXV, 63262, *RTC1* |  | chrIII, 264555, nc  chrV 33475,nc (9 bp upstream of the *CAN1* start codon) |  |
|  |  |  |  |  | chrXIII, 21958, nc |  |
|  |  |  |  |  | chrXV, 397478, protein of unknown function |  |
|  |  |  |  |  |  |  |
| I10 | E | 3 | chrIV, 238498, *CDC48* | 3 | chrXV, 721829, nc |  |
|  |  |  | chrVII, 189120, *PMR1* |  | chrI, 143575, nc (132 bp upstream of the *VPS8* start codon) |  |
|  |  |  | chrVII, 685650, *ESP1* |  | chrX, 59054, nc |  |
|  |  |  |  |  |  |  |
| I16 | H | 3 | chr IV, 1263974, *SXM1* | 4 | chrI, 35113, nc | 10 |
|  |  |  | chrVII, 189915, *PMR1* |  | chrXII, 253323, nc (538 bp upstream of the *ERG3* start codon) |  |
|  |  |  | chrXVI 454870 *LGE1* |  | chrXII, 704138, nc |  |
|  |  |  |  |  | chrXV, 1049251, nc |  |
|  |  |  |  |  |  |  |
| I16 | D | 3 | chrVII, 190466, *PMR1* | 7 | chrII, 767793 (187 bp upstream to *DUG2* start codon) |  |
|  |  |  | chrVII, 245858, *FLD1* |  | chrIX, 127338, nc (324bp upstream of the *SIM1* start codon) |  |
|  |  |  | chrVII, 1027871, *YTA7* |  | chrX, 269905, nc (90 bp downstream of the *ARG3* stop codon,  97 bp downstream of the *TRL1* stop codon) |  |
|  |  |  |  |  | chrXII, 807323 (62 bp upstream of the *SPO77* start codon) |  |
|  |  |  |  |  | chrXII, 823566, nc |  |
|  |  |  |  |  | chrXIV, 235881, *ALG9* |  |
|  |  |  |  |  | chrXV, 397478, protein of unknown function |  |
|  |  |  |  |  |  |  |

SNP and Indel mutations were identified by whole genome sequencing of one individual clone purified from each of ten independently evolved populations (Materials and Methods). The individual clones (A, B, C, etc.) are indicated as being from incompatible (I) or compatible (C) strains evolved for 10 or 16 transfers. nc indicates a SNP or indel was detected in a non-coding region.
